# Concentration Effect on Quenching of Chlorophyll a Fluorescence by All-Trans-β-Carotene in Photosynthesis

**DOI:** 10.3390/molecules22101585

**Published:** 2017-09-21

**Authors:** Chen Chen, Nan Gong, Zuowei Li, Chenglin Sun, Zhiwei Men

**Affiliations:** 1 Coherent Light and Atomic and Molecular Spectroscopy Laboratory, College of Physics, Jilin University, Changchun 130012, China; chenchen16@mails.jlu.edu.cn (C.C.); gongnan15@mails.jlu.edu.cn (N.G.); zuowei_li@163.com (Z.L.); 2 Key Laboratory of Physics and Technology for Advanced Batteries, College of Physics, Jilin University, Changchun 130012, China

**Keywords:** Chl-a, fluorescence, β-Car, quenching

## Abstract

Absorption, fluorescence spectra of chlorophyll a (Chl-a) and all-trans-β-carotene (β-Car) mixing solution are investigated in different polarity and polarizability solvents. The carotenoids regulate the energy flow in photosynthesis by interaction with chlorophyll, leading to an observable reduction of Chl-a fluorescence. The fluorescence red shifts with the increasing solvent polarizability. The energy transfer in the Chl-a and β-Car system is proposed. The electron transfer should be dominant in quenching Chl-a fluorescence rather than the energy transfer in this system. Polar solvent with large polarizability shows high quenching efficiency. When dissolved in carbon tetrachloride, Chl-a presents red shift of absorption and blue shift of fluorescence spectra with increasing β-Car concentration, which implies a Chl-a conformational change.

## 1. Introduction

Photosynthesis is the biological basis of most life on Earth, converting sunlight to biomass, which is of great importance for the growth and development of plants. Chlorophyll and carotenoids are both building blocks of the photosynthetic apparatus [1,2,3,4,5,6]. Chl-a can be found in higher plants, green algae, and some bacteria, and is considered the major light-harvesting pigment in these organisms [7,8,9,10]. Carotenoids are alkene molecules that contain π-electron conjugated bonds, and have many functions in organic life, such as energy transfer, photoprotection and interception of singlet oxygen [11,12,13]. Carotenoids interacting with chlorophyll in the singlet state which will lead to a quenching of chlorophyll fluorescence. This indicates their key role in regulating the energy flow of chlorophyll excited states. Studies have shown that β-Car can quench Chl-a fluorescence in organic solvents [14]. Several studies suggest that different solutions surrounding the photosynthetic system will lead to aggregation or other structural changes inducing the photophysical changes. This means that the solution should be added with caution for photosynthetic systems. The molecular mechanism of dissipation process still remains under debate, although several models have been proposed [3,5].

This work investigates the fluorescence of Chl-a characteristics quenched by β-Car. The concentration is around 10^−5^ M, the quenching efficiency improves with the increasing concentration. Solvent polarizability effects on quenching are monitored by the position of the absorption of β-Car and the fluorescence position of Chl-a. The physical interaction mechanism of β-Car and Chl-a is illustrated.

## 2. Results and Discussion

**Figure 1:**
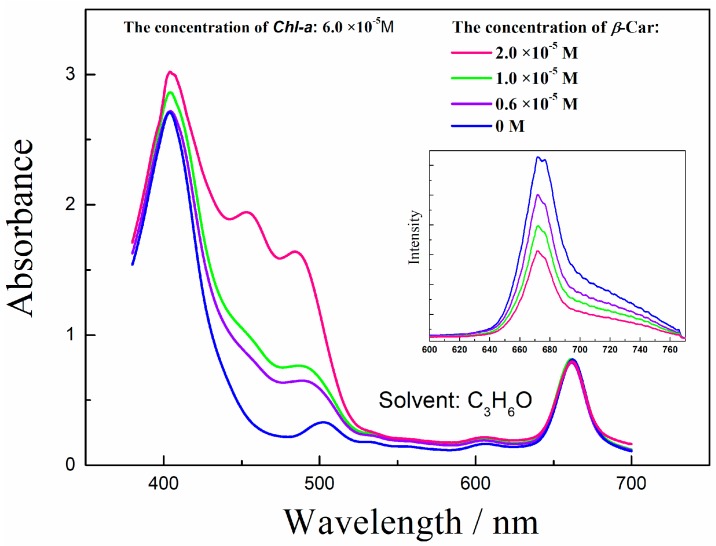
Absorption spectra of Chl-a and β-Car mixing solution, inset: the corresponding Chl-a fluorescence emission. The excited wavelength of the emission is 532 nm.

Individual Chl-a solution shows two strong absorption bands located at 640~670 nm and 410~430 nm. The absorption peaks of β-Car show three obvious vibronic peaks, and belong to the transition S_0_ → S_2_. The three peaks are the phonon assist band and are labelled, from left to right, 0-2, 0-1 and 0-0. In the Chl-a and β-Car mixing solution, the 0-2 absorption band overlaps with the Chl-a 410 nm band (Figure 1).

The mixing solution shows overlap of the absorption spectra of Chl-a and β-Car. When the β-Car concentration is increased, the 0-0 peak shows a blue shift, which leads to a much greater spectral overlap with the absorption of Chl-a. The superposition between Chl-a and β-Car 0-2 peaks enhances the absorption at 410 nm. Simultaneously, the fluorescence decreases with the increasing concentration.

**Figure 2:**
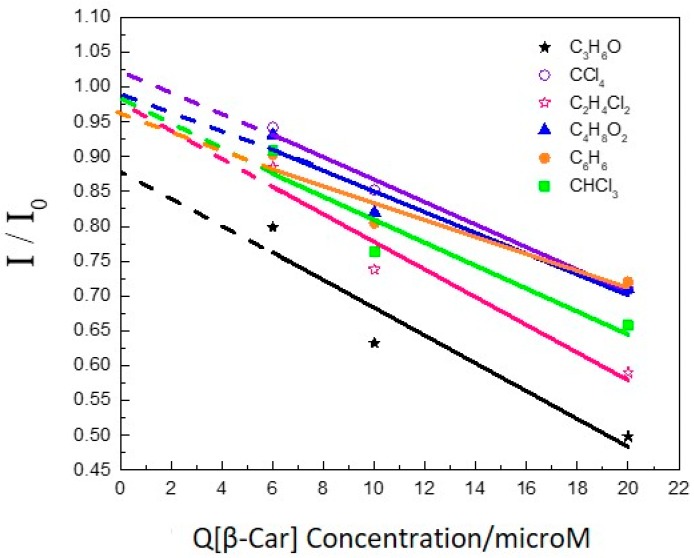
Stern-Volmer type plot of Chl-a and β-Car mixing in different solvents.

Figure 2 presents the fluorescence intensity variation after adding β-Car. The acetone solution shows the highest quenching efficiency, due to its large dielectric constant, which is somewhat larger than 20. Large quantum efficiency is observed in polar solvent because the solute tends to aggregate. Thus, the photophysical properties will vary in solvents with different polarities. Moreover, polar solvents are analogous to adding an electric field among the molecules, which generates the intrinsic dipole moment. With intrinsic dipole moments, intermolecular electrostatic interaction will occur between two molecules. Upon the interaction of an excited singlet state of β-Car with Chl-a, the energy can be partitioned between energy transfer and an electron transfer process [14]. Since β-Car has a lower ionization potential, the electron transfer should predominate in the center of reaction. Through fast interaction between β-Car ground state and Chl-a excited state, a non-emissive exciplex intermediate is produced. β-Car^+^ forms and a rapid ground state reaction appears with neighbor chlorophyll molecules. As a result, Chl−·Chl+ is produced, and this dimer species is involved in photosystem II [15]. Among the different solvents, we have about 50% quenching or more at a carotene concentration of 2 × 10^−5^ M. Assuming dynamic quenching, this corresponds to a Stern Volmer constant of 5 × 10^4^ M^−1^. For a singlet decay time of the chlorophyll of 10^−8^ s, this would correspond to a fluorescence quenching rate constant of 5 × 10^12^ M^−1^ s^−1^. Actually, 10^−8^ s is really an upper limit for the singlet decay time, which is in fact 10^−9^ s making the fluorescence quenching rate constant 5 × 10^13^ M^−1^ s^−1^. This is two to three orders of magnitude higher than the rate constant for a diffusion-controlled process, which is the maximum rate constant possible for fluorescence quenching by a short-range process as Dexter transfer electron transfer. For the same reason, we can also exclude the dynamic quenching by Förster transfer, as this would correspond to an unrealistic R_0_ value of 8 to 80 nm. Additionally, static Förster transfer to randomly distributed carotenes can be excluded. The average distance to a carotene is about 25 nm at a concentration of 2 × 10^−5^ M. This is much larger than any value reported for R_0_, especially considering that the S_0_ → S_1_ transition in carotene is symmetry forbidden. Therefore, we must conclude that the carotene and chlorophyll form a complex, and that inside this complex, very efficient quenching of the chlorophyll singlet by electron transfer of Dexter type energy transfer occurs [16,17,18].

**Figure 3:**
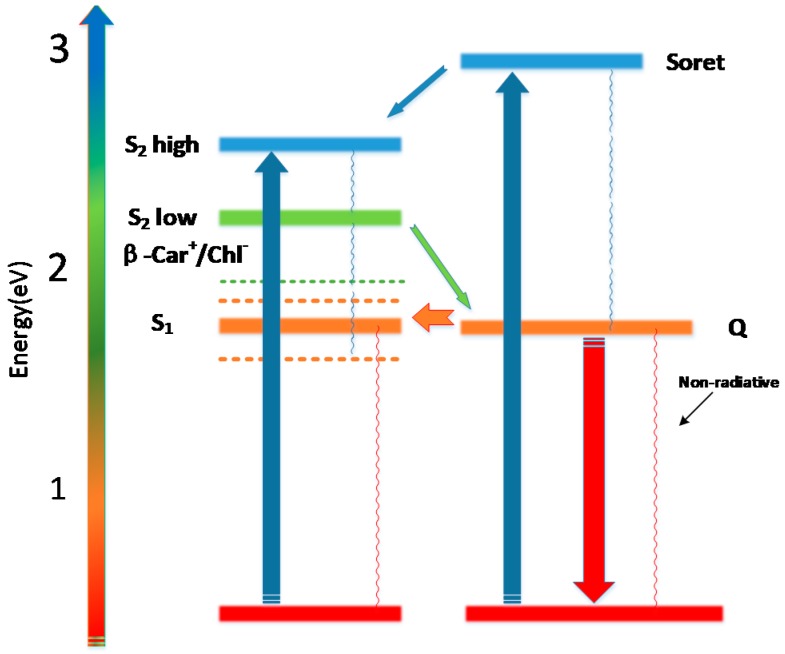
Proposed energy transfer scheme in Chl-a and β-Car in different solvents.

Direct energy transfer from carotenoid was traditionally considered impossible, because the energy of the excited singlet state of Chl-a lies below that of β-Car. It is suggested the symmetry forbidden S_0_ → S_1_ transition leads to a small transition dipole moment. Therefore, the Förster type energy transfer seems to be inappropriate, and the Dexter electron exchange mechanism would be more appropriate. However, recent reports of a symmetry effect on carotenoids shows that even the β-Car in solution should not have the C_2h_, but C_2_ should be the proper symmetry group [4]. There is no symmetry-forbidden electronic transition in β-Car. The S_1_ state of β-Car lies on the ~4000 cm^−1^ lower in energy than that of S_2_ [18]. If the number of carbon-carbon double bonds exceeded 10, the S_1_ state would lie below that of Chl-a. In this context, energy transfer could appear in the Chl-a and β-Car system, and the β-Car would be able to act as a trap for the dissipation of excess energy. The proposed energy transfer scheme in Chl-a and β-Car in different solvents is shown in Figure 3.

**Figure 4:**
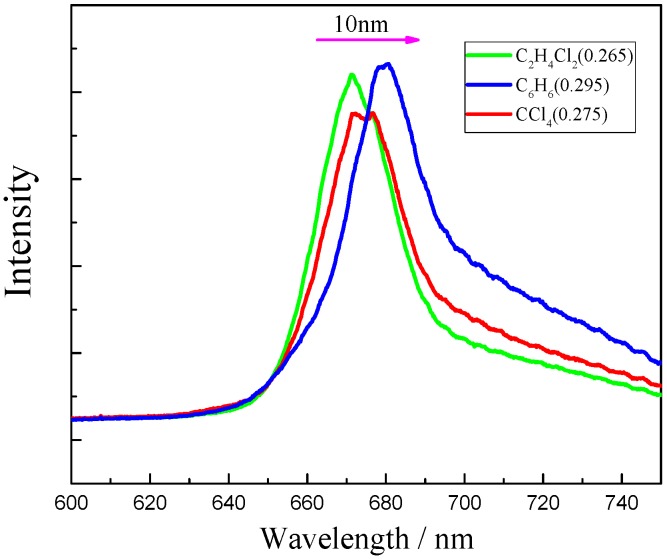
Fluorescence spectra red-shift by increasing solvent polarizability.

**Figure 5:**
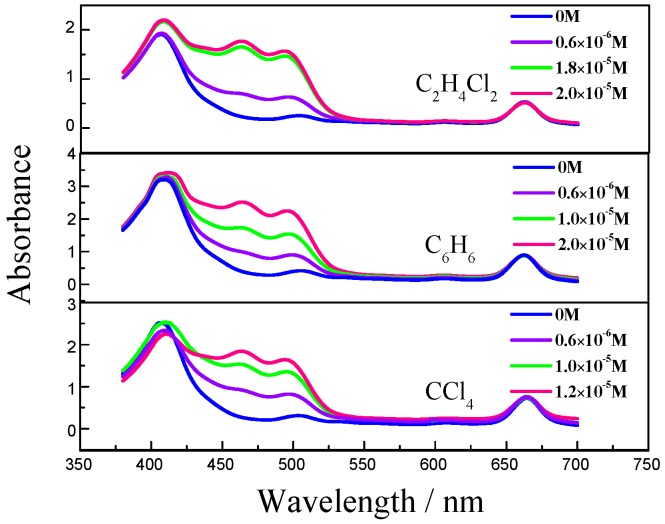
Absorption spectra of Chl-a and β-Car in different polarizability non-polar solvents.

Another factor that should be taken into account in the quenching process is solvent polarizability. Figure 4 and Figure 5 show the Chl-a and β-Car absorption and fluorescence versus solvent polarizability. Interestingly, both spectra show a red-shift following the solvent polarizability increase. With an increase in solvent polarizability, the fluorescence position of the Chl-a red-shift is about 10 nm. The absorption spectra of the Chl-a and β-Car system in non-polar solvent are shown in Figure 5. Neither the Chl-a nor β-Car absorption peaks show a particularly large shift. Considering that the β-Car S_1_ state is more solvent-sensitive, the red-shift versus solvent polarizability will be relatively large. The β-Car S_1_ state is the route of the energy transfer, and red-shifts with increasing solvent polarizability. Therefore, energy transfer should make a contribution, and is dependent on molecular distance. The environment in antennae is known to be different from that of the reaction center, and energy transfer is relative to the environment of the carotenoids [19]. Absorption and vibrational techniques have shown that polarizability is an important environmental parameter both in vivo and in vitro. Conformational change correlates significantly with the conjugation length of carotenoids. This means that carotenoids in different polarizability environments should have different roles in regulating energy flow. We suggest that both electron transfer and energy transfer contribute to quenching Chl-a fluorescence in vitro. Moreover, polar environments with greater polarizability are considered to lead to higher quenching efficiency.

**Figure 6:**
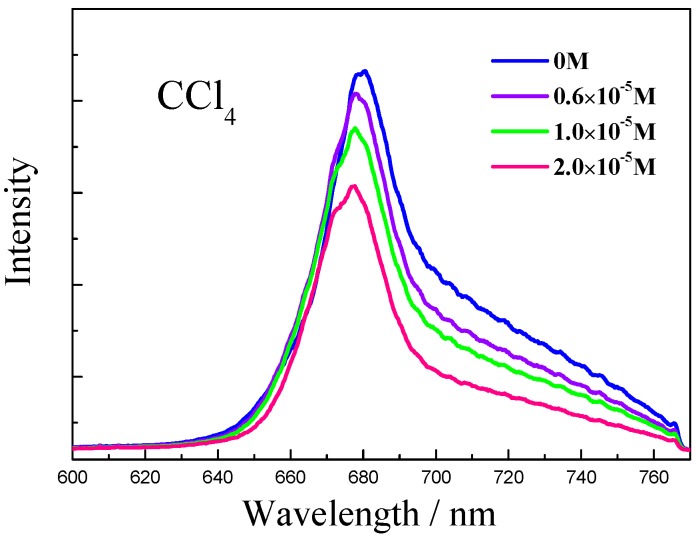
Fluorescence blue-shift with increasing concentration of β-Car.

Unlike other observations in this work, Chl-a in CCl_4_ solvent undergoes a red-shift of absorption and a blue-shift of fluorescence (Figure 5 and Figure 6). The red shift of absorption implies conformational change of the Chl-a. Two effects should be taken into account: One is the out-of-plane distortion effect between Chl-a and β-Car mixing molecules and CCl_4_, the other is the laser-heating induced conformational change [20,21]. The blue shift of fluorescence implies an enhanced population in higher vibrational level, which leads to increase of the radiation photon energy.

## 3. Materials and Methods

Chl-a powder was purchased from Bomei (Hefei, China) and stored under cold and dark condition before use. The purity was checked by comparing its absorption bands. β-Car from Sigma Aldrich (Shanghai, China) was used without further purification. All samples contained the same Chl-a with the same concentration at 10^−5^ M. The concentration gradients of β-Car were 0.6, 1.0 and 2.0 × 10^−5^ M, respectively. Spectrograde solvents were used. The fluorescence of Chl-a was measured at ~670 nm perpendicular to the incident light, and was recorded by Ocean Optics Maya 2000 (Ocean Optical Corporation, Dunedin, FL, USA). The excited wavelength was 532 nm. The detector was fixed perpendicular to the quazt cell at an appropriate distance to record the fluorescence. Absorption spectra were measured on a double-beam UV-Vis spectrophotometer TU-1901 (Persee Co., Ltd., Beijing, China) with a step of 0.5 nm at room temperature.

## 4. Conclusions

In conclusion, absorption and fluorescence show an effective quencher role of β-Car. The Chl-a fluorescence is reduced by increasing the concentration of β-Car. Increasing the solvent polarizability leads to a red-shift of the fluorescence of Chl-a. The results suggest that electron transfer dominates in the Chl-a and β-Car system. Different solvent environments determine the relative importance of these two processes, which are related to the distance between the molecules. Polar solvents with high polarizability show a higher quenching efficiency. On excitation in CCl_4_, a red shift of Chl-a absorption and a blue shift of fluorescence are observed. All these results lead to a strong environmental effect on quenching Chl-a fluorescence. More experimental studies will be conducted on this issue and the mechanism of quenching of Chl-a fluorescence in photosynthesis.
